# Novel pencil-beam scanning proton lattice radiation therapy for the treatment of bulky liver cancer: dosimetric comparison with VMAT-lattice radiotherapy

**DOI:** 10.3389/fonc.2025.1731259

**Published:** 2026-02-04

**Authors:** Yuting Li, Rachael M. Martin-Paulpeter, Christine V. Chung, Miguel A. Aguilar, Carlos A. Matias, Adrian Delgado, Saurabh S. Nair, Laurence E. Court, Luis Perles, Narayan Sahoo, Ronald X. Zhu, Falk Poenisch, David B. Flint, Gabriel O. Sawakuchi, Sam Beddar, Eugene Koay, Jamie S. Baker, Ethan B. Ludmir, Joshua S. Niedzielski

**Affiliations:** 1 Department of Radiation Physics, The University of Texas MD Anderson Cancer Center, Houston, TX, United States; 2 Graduate School of Biomedical Sciences, University of Texas Health Science Center at Houston/The University of Texas MD Anderson Cancer Center, Houston, TX, United States; 3 School of Health Professions, The University of Texas MD Anderson Cancer Center, Houston, TX, United States; 4 Department of Gastrointestinal Radiation Oncology, The University of Texas MD Anderson Cancer Center, Houston, TX, United States

**Keywords:** bulky tumor, hepatocellular carcinoma, lattice radiation therapy, liver cancer, peak-to-valley dose ratio, proton therapy, VMAT, spatially fractionated radiation therapy

## Abstract

**Background:**

Spatially fractionated radiotherapy (SFRT) delivered as lattice radiotherapy (LRT) creates high-dose ‘vertex’ subvolumes (VTVH) embedded within low-dose ‘valley’ regions (VTVL) to intensify intratumoral dose while minimizing radiation dose and toxicity to organs at risk (OARs). We developed a pencil-beam scanning (PBS) intensity modulated proton therapy (IMPT) LRT planning approach and dosimetrically compared it directly with contemporary photon-based volume modulated arc therapy (VMAT) LRT for liver cancer with bulky tumors.

**Methods:**

Twenty-one retrospective liver cases were replanned in RayStation to a single fraction of 20 Gy to VTVH with explicit VTVL sparing (mean dose <5Gy). Primary dosimetric endpoints were VTVH D80 and VTVL mean dose (analogous to mean valley dose), with secondary endpoints of VTVH/VTVL ratios (PVDR-like metrics at D80/D90/D100), VTVL D5/D80, GTV D10/D90, and planning risk volume (PRV) 0.03 cm³ hotspots. Statistical analysis consisted of paired tests (t-test or Wilcoxon signed-rank) after assessing data normality (Shapiro-Wilk tests) and using α=0.05 for significance.

**Results:**

Compared with photon VMAT-LRT, IMPT-LRT significantly reduced VTVL mean dose (p<0.00001) and increased VTVH D80 (p<0.00001), yielding higher VTVH/VTVL ratios at D80/D90/D100 (all p<0.00001). Gross tumor volume (GTV) heterogeneity (D20/D80) significantly increased with proton-LRT (p<0.00001) and OAR hot-spot metrics (PRV D0.03cc) were comparable between both modalities (p=0.71).

**Conclusions:**

A robust PBS proton-LRT planning approach was developed and compared to photon VMAT-LRT for large liver tumors. Our IMPT-LRT approach maximizes peak-to-valley separation and simultaneously maintains target coverage while significantly reducing valley dose, as compared to traditional VMAT-LRT. These findings support future prospective clinical evaluation.

## Introduction

Liver cancer presents great challenge for radiation therapy (RT) due to the desired preservation of liver function, tumor proximity to critical organs, and motion uncertainties due to respiration and peristalsis; these confounding factors are further amplified when in the setting of bulky liver tumors (1–3). Radiation dose escalation is limited due to the risk of radiation-induced liver disease (RILD) and the need to spare adjacent gastrointestinal organs, which often leads to conservative dose constraints even with utilization of advanced imaging and motion management techniques during radiation delivery (1, 4). Maintaining functional hepatic reserve is a primary determinant of safety. Classic NTCP/QUANTEC analyses link mean liver dose and spared liver volume to the risk of classic RILD and contemporary guidelines for primary liver cancers recommend preserving a critical volume of uninvolved liver (e.g., ≥700 cm³ kept below 15 Gy for 3 fractions or 21 Gy for 5 fractions in SBRT schemas) and constraining mean liver dose within 28 Gy or 32 Gy depending on the diagnosis, particularly in cirrhotic patients (1, 5, 6). These realities motivate development of radiation therapy modalities that would intensify tumor dose while maintaining mean liver dose and meeting organ-at-risk (OAR) dose constraints.

Spatial Fractionated Radiotherapy (SFRT) has re-emerged as an advanced radiation therapy approach that is particularly well-suited to treat deep-seated lesions (7). Rather than aiming for uniform target coverage, SFRT intentionally delivers a spatially modulated dose with alternating high-dose “peaks/vertices” and low-dose “valleys,” while maintaining OAR dose within tolerance. Historically, GRID, one types of SFRT, used a physical GRID block to create a 2D pattern (8); one drawback to GRID is that the 2D treatment approach is not well suited for deep-seated tumors due to rapid dose falloff as a function of depth. Subsequent advances in multi-leaf collimation and inverse planning has enabled the wide-spread adoption of lattice radiotherapy (LRT), another form of SFRT in which spherical high-dose vertices are distributed throughout the gross tumor volume (GTV) in a lattice pattern, thereby enabling advantageous dose distribution within deep-seated tumor, such as bulky liver cancer (7, 9, 10). Current publications about LRT have established general consensus about vertex coverage, valley dose, peak-to-valley dose ratio (PVDR), and planning approaches, which has enabled the clinical translation of LRT (11, 12).

LRT is typically utilized using volume modulated arc therapy (VMAT) on standard photon-based linear accelerators and has demonstrated its capability of creating high intratumoral vertices and meeting OAR dose constraints (13, 14). However, exit dose and integral dose remain intrinsic to photons, and maintaining low valley dose becomes progressively more challenging as tumor size increases, potentially limiting the extent of dose escalation or fraction numbers achievable in the setting of photon LRT (12, 14–16).

Proton therapy offers dosimetric advantage over photon-based RT due to its sharp distal fall-off, the so-called Bragg peak, which eliminates beam exit dose, as well as overall integral dose. Given the potential immune modulating advantages of SFRT, reduction of integral dose in the tumor volume is a challenge in photon-based SFRT (17, 18). Naturally, this raises the hypothesis that proton-based LRT could significantly lower valley dose and improve PVDR-like metrics relative to photon-based LRT, particularly for large hepatic targets.

To elucidate the potential utility of proton-based LRT in the setting of bulky liver cancers, we developed a practical treatment planning strategy to implement LRT with intensity modulated proton therapy (IMPT) using a clinical pencil beam scanning (PBS) proton beam line. We also performed a paired dosimetric comparison of IMPT-LRT with photon VMAT-LRT in a retrospective cohort of liver cancer patients with bulky disease. The aim of this work was to determine if IMPT-LRT is superior in minimization of valley dose and maximization of PVDR dose characteristics, while maintaining peak vertex dose coverage, as compared to standard photon-based VMAT-LRT, under a common clinically prescribed, single-fraction 20Gy LRT treatment (11, 12, 16, 19, 20).

This work contributes three elements for the clinical SFRT community: (i) a reproducible, liver-specific IMPT-LRT planning workflow with robust optimization; (ii) quantitative, paired evidence against a standard-of-care VMAT-LRT implementation using the same lattice geometry; and (iii) a reporting framework anchored to consensus SFRT/LRT metrics to facilitate cross-study comparison and protocol development.

## Materials and methods

### Study design and cohort

We performed a retrospective planning study of 21 large liver cancer cases. CT datasets and structure sets were collected from previous conventional RT clinical plans, which have been contoured and approved by the treating radiation oncologist. All 21 study patients were previously treated with breath-hold (BH) motion management technique to minimize respiratory motion during RT. For each patient, identical planning CT and structure sets were used to ensure consistency between IMPT and VMAT LRT plans. A single-fraction prescription of 20 Gy to the high-dose lattice vertices (Vertex Tumor Volume-High, VTVH) was utilized and plans were reviewed for clinical acceptability by clinical physicists and physicians.

### Lattice creation and clinical goals

**Figure 1:**
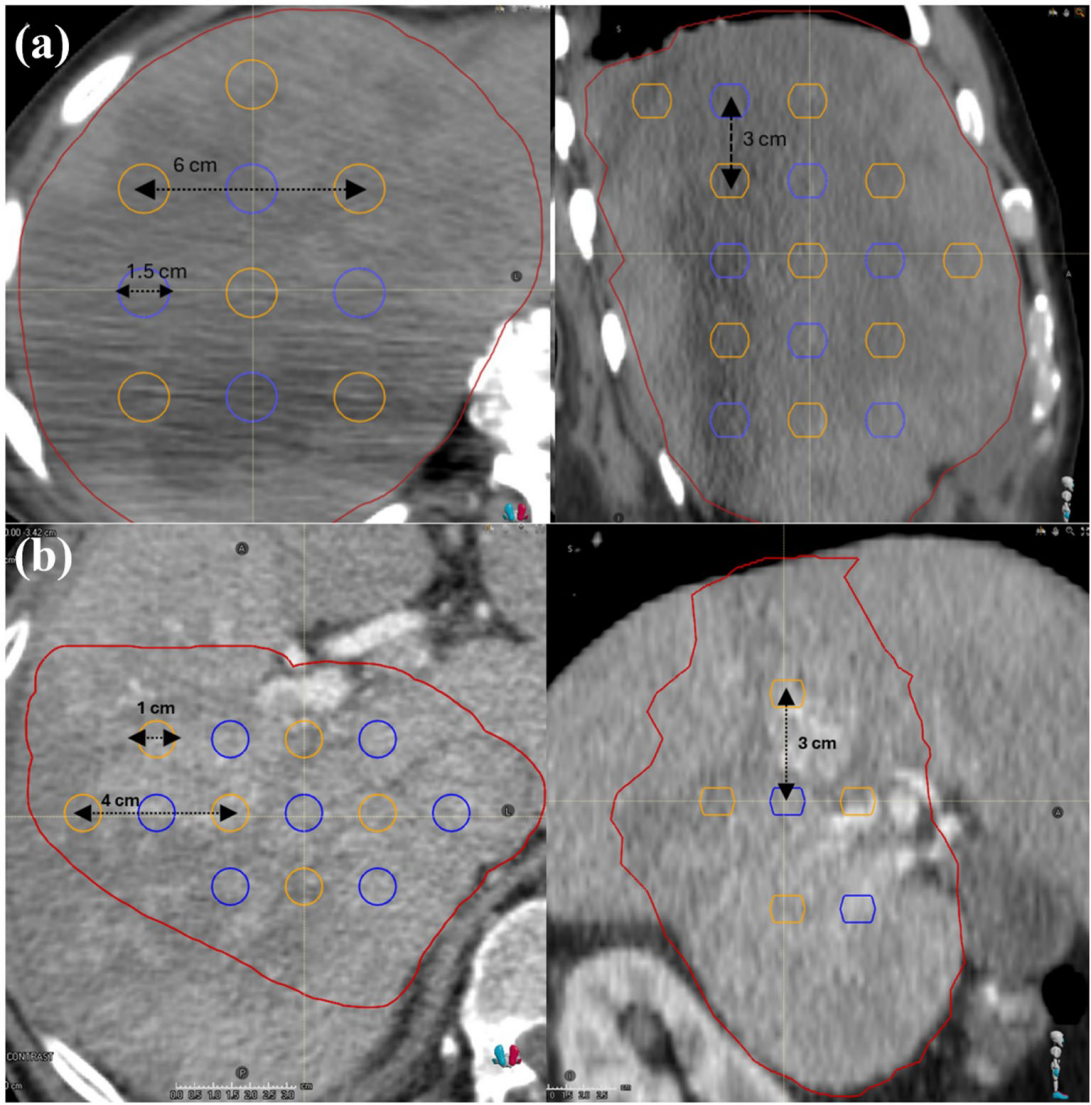
Lattice construction for tumor volume under and over 1000 cm³, where high-dose vertices (VTVH) are shown in blue, and low dose avoidance vertices (VTVL) are shown in orange. Sphere creation and layout for tumors with a gross tumor volume of **(a)** greater than or equal to 1000 cm³, and **(b)** less than 1000 cm³.

A script in RayStation has been developed to generate spherical prescriptive VTVH (high-dose vertices) and vertex tumor volume-low (VTVL, valley dose avoidance structure) within the radiation oncologist contoured clinical GTV. 1 cm margin was given to the edge of GTV and from OARs when constructing the lattice structures. Lattice characteristics (i.e., vertex size and spacing) conformed to a standard protocol according to GTV size. For patients with GTV <1000 cm^3^, 1-cm diameter spheres with 3 cm superior-inferior and 4 cm lateral center-to-center (CTC) spacing were created. For cases with GTV ≥1000 cm^3^, sphere diameters increased to 1.5 cm with 3-cm and 6-cm superior-inferior and lateral CTC spacing, respectively. This lattice placing strategy was established during planning study for VMAT cases, which would guarantee the optimal PVDR. Same lattice arrangement is used for each patient in VMAT and IMPT cases. Two examples of lattice construction are shown in **Figure 1**. Planning dose constraints and objectives are listed in **Table 1**.

**Table 1:** Planning dose constraints.

Metric	Constraints
VTVH Dmean	≥ 20 Gy
VTVH D80	≥ 20 Gy
VTVL Dmean	≤ 4 Gy
VTVL D5	≤ 5 Gy

### VMAT-LRT planning

VMAT-LRT plans were created in our institution’s clinical TPS (RayStation, RaySearch Laboratories; Stockholm, Sweden) using a 6FFF beam energy with 3–5 full arcs, multiple collimator angles (0°, 15°, 30°, and/or 90°) and a Truebeam (Varian Medical Systems; Palo Alto, CA) beam model with collapsed cone convolution dose algorithm and 2mm dose grid. Optimization objectives prioritized VTVH coverage and VTVL sparing while meeting OAR limits. OAR constraints consisted of heart max dose <8 Gy, right kidney max dose <10Gy and PRV max dose <8 Gy. The OAR_PRV structure was defined as the concatenation of bowel, stomach, duodenum, and esophagus, plus a 5-mm expansion margin.

### IMPT-LRT planning

**Figure 2:**
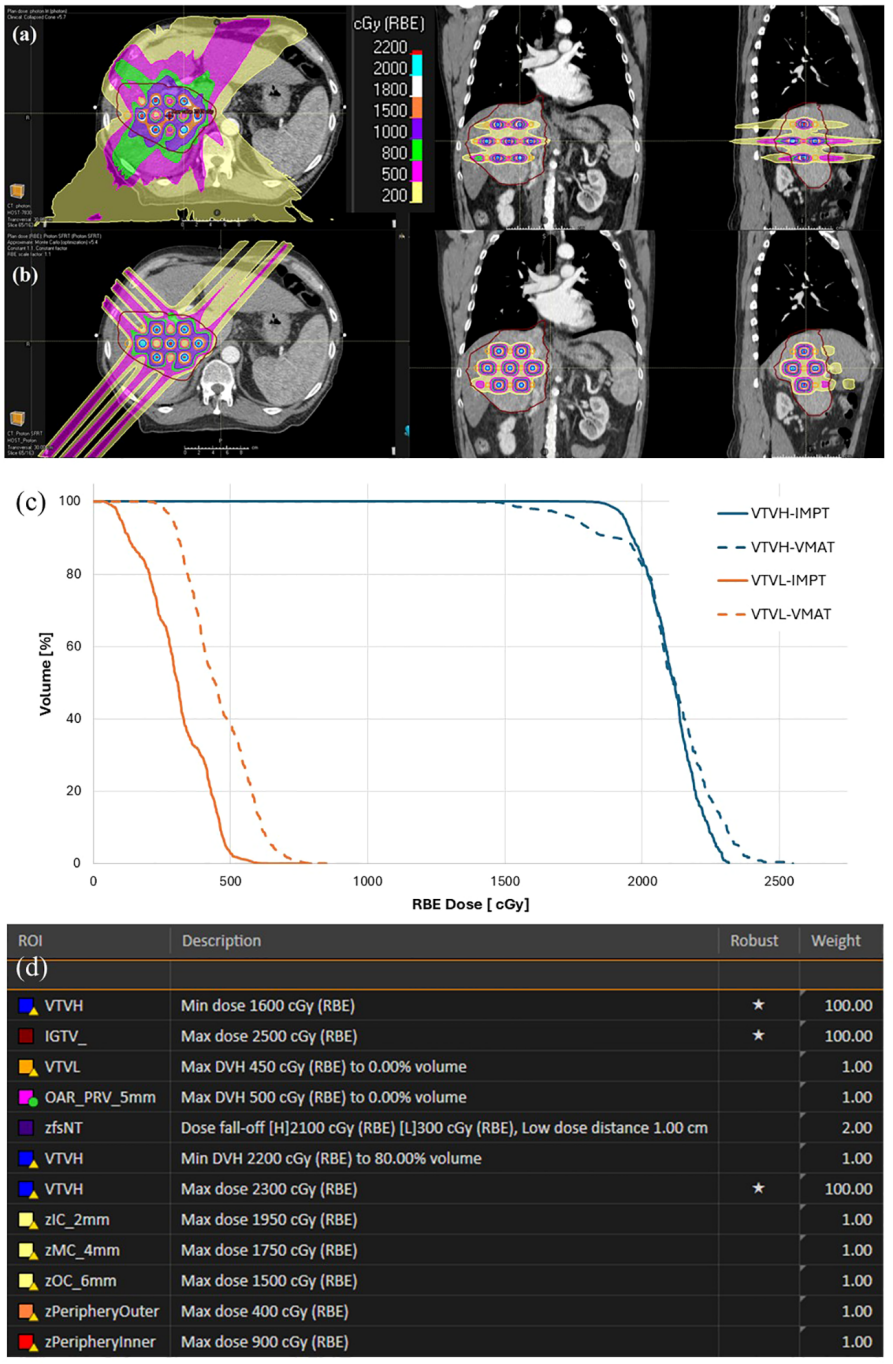
Example patient comparison of dose distributions and DVH for VMAT-LRT and IMPT-LRT. Axial, coronal, sagittal dose distributions illustrating dose distribution in a **(a)** VMAT-LRT and **(b)** IMPT-LRT. **(c)** DVHs for VTVH and VTVL comparing VMAT-LRT and IMPT-LRT. **(d)** Optimization objectives used in IMPT-SFRT planning. Besides VTVH, VTVL, IGTV and fsNT, other structures are ring structures to control the dose fall off.

Proton lattice plans were created with the clinical beam model for Hitachi (Hitachi Ltd. Tokyo, Japan) ProBeat proton delivery system in RayStation. PBS dose calculation was done with RayStation Monte Carlo model and 2-mm dose grid, which is the same as the corresponding VMAT plan. Hitachi ProBeat is a synchrotron based, 4-room treatment system, where proton beam with 83 discrete energies ranging from 70.2 MeV to 228.7 MeV can be generated. The same structure sets (clinical GTV, lattice ROIs, and OARs) were used for planning as the VMAT-LRT plans. The identical lattice ROIs (i.e., VTVH and VTVL) and locations were used for both VMAT-LRT and IMPT-LRT plans. For every patient, two anterior oblique and one posterior oblique beams along the vertex lines of lattice were used, and beam arrangements aimed to avoid directly opposing beams, and maximize VTVH coverage and to minimize both VTVL and OAR doses. A range shifter was not used in planning due to the non-superficial nature of lattice target structures, where the 83 discrete energies can cover the WET varying from 4cm to 32.4cm. Energies per plan were selected automatically by the optimizer based on the size and depth of the vertices, 5-mm spot spacing was used in each energy layer, and dose repainting was used to minimize the interplay effect for IMPT-LRT delivery (4). Robust optimization included 3.5% range and 3-mm setup uncertainties. A constant 1.1 RBE was used for proton plans. Robust min DVH objective was used for VTVH to achieve vertex coverage and conformity, and various robust max DVH objectives were used for VTVL to achieve dose sparing. Iterative approach was taken for each plan to achieve the desired PVDR. We have included the optimization objectives in one IMPT-SFRT plan in **Figure 2** for reference.

### Endpoints and statistics

Primary endpoints were VTVH D80 and VTVL mean dose (analogous to mean valley dose). Secondary endpoints included, VTVH/VTVL ratios (PVDR-like metrics at D80/D90/D100), VTVL D5/D80, GTV D20/D80, and OAR_PRV 0.03 cm³ hotspots. Normality of paired differences was assessed using Shapiro–Wilk. If normality was not rejected, we used paired t-tests; otherwise, Wilcoxon signed-rank tests (two-sided, α=0.05). For multiple endpoints, the familywise error rate was controlled via Holm adjustment; the primary endpoint is specified in the Results. We report mean ± SD for endpoints analyzed by paired t-test and median (min–max) for those analyzed by the Wilcoxon signed-rank test; exact p-values are provided in **Tables 2**–**4**.

**Table 2:** VTVL and PRV dose metrics and statistical comparison between VMAT-LRT and IMPT-LRT.

Dose metric	VMAT-LRT (Gy)	IMPT-LRT (Gy)	P value
Mean Dose	3.56 (2.98-4.58)	1.89 (0.81-3.04)	1.41E-09^†^
VTVL D5	4.89 (4.05-7.47)	3.76 (2.13-4.76)	1.91E-06^†^
VTVL D50	3.47 (2.83-4.43)	1.78 (0.56-3.03)	5.03E-07^†^
VTVL D80	2.91 (2.28-3.45)	0.91 (0.15-2.04)	8.90E-08*
VTVL D90	2.67 (1.93-3.16)	0.58 (0.09-1.35)	2.10E-08*
VTVL D100	1.80 (1.13-2.37)	0.13 (0.01-0.44)	4.55E-11*
PRV 0.03cm^3^	6.66 (3.27-10.20)	5.75 (0.02-9.59)	0.071 ^†^

a) ^†^Paired t-test performed; *Paired Wilcoxon rank test performed.

b) Mean (min-max) dose.

**Table 3:** VTVH dose metrics and statistical comparison between VMAT-LRT and IMPT-LRT.

Dose metric	VMAT-LRT (Gy)	IMPT-LRT (Gy)	P value
VTVH D5	24.65 (23.52-26.02)	23.47 (22.37-24.46)	8.79E-07^†^
VTVH D50	21.93 (21.21-22.94)	22.17 (21.14-22.88)	1.40E-05^†^
VTVH D80	20.37 (20.00-21.34)	21.24 (20.11-22.05)	9.00E-04*
VTVH D90	19.22 (17.88-20.64)	20.69 (19.68-21.60)	0.062*
VTVH D100	10.85 (6.83-16.43)	16.32 (10.67-18.91)	2.38E-05^†^
VTVH/VTVL D80	8.36 (5.82-33.37)	38.42 (9.86-123.47)	1.91E-06*
VTVH/VTVL D90	8.81 (6.06-37.90)	64.86 (15.07-204.60)	9.99E-08*
VTVH/VTVL D100	7.48 (3.83-33.23)	360.48 (41.57-1844)	7.25E-07*

a) ^†^Paired t-test performed; *Paired Wilcoxon rank test performed.

b) Mean (min-max) dose.

**Table 4:** GTV coverage and statistical comparison between VMAT-LRT and IMPT-LRT.

Dose metric	VMAT-LRT (Gy)	IMPT-LRT (Gy)		P value
GTV D5	10.78 (8.47-13.52)	14.08 (10.61-17.61)		3.60E-10*
GTV D10	8.05 (6.17-10.23)	9.85 (6.99-12.46)		6.92E-08*
GTV D20	5.55 (4.12-7.54)	6.00 (3.75-8.30)		0.023*
GTV D50	2.32 (1.28-3.84)	1.51 (0.32-3.46)		0.000194*
GTV D80	1.09 (0.29-2.22)	0.16 (0.03-0.63)		9.54E-07^†^
GTV D90	0.60 (0.19-1.43)	0.06 (0.01-0.22)		9.54E-07^†^
GTV D100	0.15 (0-0.42)	0.02 (0-0.02)		1.91E-06^†^
GTV D20/D80	5.10 (3.40-14.21)	55.14 (303.60-946)		9.54E-07^†^

a)^†^Paired t-test performed; *Paired Wilcoxon rank test performed.

b) Mean (min-max) dose.

## Results

### VTVL (valley) sparing and VTVH (peak) coverage

**Figure 3:**
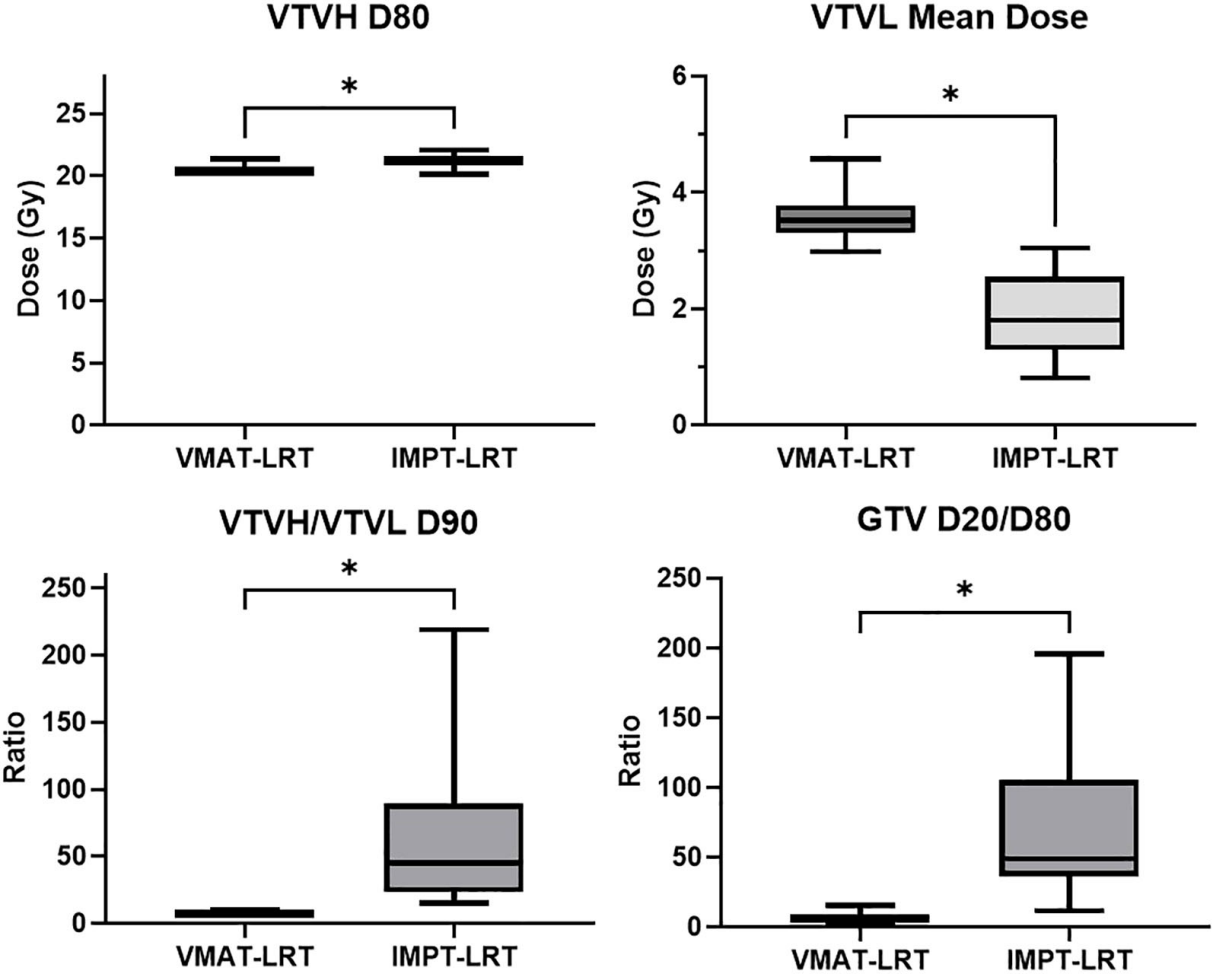
Box-and-whisker plots for paired comparisons of VMAT-LRT and IMPT-LRT for **(a)** VTVH D80, **(b)** VTVL mean dose, **(c)** ratio of D90 for VTVH and VTVL, and **(d)** ratio of GTV D20 and D80. Paired statistical comparisons show significant differences in dosimetric values between the two planning modalities, with * indicating P<0.05.

A comparison of IMPT-LRT and VMAT-LRT dose distributions and dose-volume histogram (DVH) metrics for VTVL and VTVH are shown for a sample patient from the 21 patient study cohort in **Figure 2**. An overall trend of higher VTVH target coverage and lower VTVL valley doses is seen in IMPT-LRT plans, when compared to VMAT-LRT plans, as shown in **Figure 3**.

VTVL dose metrics and paired statistical analysis results are listed in **Table 2**. For all the 21 patients studied, IMPT−LRT achieved a consistent and statistically significant reduction in VTVL mean dose compared with VMAT−LRT (subgroup averages, 1.89 Gy vs. 3.56 Gy; p=1.41E-09). This trend of lower VTVL doses from IMPT-LRT, as compared to VMAT-LRT, was consistent for all metrics examined (i.e., VTVL D5, VTVL D50, VTVL D80, VTVL D90). Qualitatively, intratumoral low−dose corridors were better preserved between vertices for IMPT−LRT, along with reduced exit and integral dose.

VTVH dose metrics and paired statistical analysis results are listed in **Table 3**. Not only did IMPT-LRT plans show a significant reduction of valley dose (VTVL), but lattice target coverage (VTVH) was either consistent between both LRT approaches, or even significantly improved by IMPT-LRT for some dose metrics (i.e., VTVH D80, VTVH D90, VTVH D100; **Table 3**). For example, IMPT−LRT increased vertex coverage over VMAT-LRT, as measured by VTVH D80 (subgroup averages, 21.24 Gy vs. 20.37 Gy; p=0.000024), without compromising OAR acceptability under the same lattice design. This translated into higher VTVH/VTVL dose−ratio metrics (PVDR surrogates) at D80, D90, and D100 (all p<0.000001), indicating stronger peak−to−valley separation across a range of isodose percentiles for IMPT-LRT.

### Global target heterogeneity

The paired statistical analysis for GTV coverage is listed in **Table 4**. Gross tumor dose heterogeneity (GTV D20/D80) increased with IMPT−LRT when compared to VMAT-LRT (subgroup average ratios, 55.14 vs. 5.10; p=9.54×10^-7^). In the lattice context, greater heterogeneity reflects more intense vertices embedded within preserved valleys, which is a desirable property when valley dose is simultaneously reduced.

### Plan acceptability and qualitative review

All plans met the single−fraction lattice goals (VTVH D80 ≥ 20 Gy, VTVL mean dose ≤ 4 Gy; VTVL D5 ≤ 5 Gy) while satisfying site−specific OAR limits during physics review. Dose displays in representative cases illustrated clearer separation between vertex peaks and valleys in IMPT−LRT, as shown in **Figure 2**.

## Discussion

This study establishes a practical PBS IMPT−LRT planning strategy for large liver tumors and demonstrates that IMPT−LRT can simultaneously deepen valleys and strengthen vertex coverage in a paired comparison with contemporary VMAT−LRT under a single−fraction 20 Gy template. The dosimetric advantages we observed are aligned with the physical properties of protons due to its minimal exit dose and lower integral dose, and with SFRT theory that prioritizes low valley dose and robust PVDR−like metrics for therapeutic index.

While we examined proton LRT in this study, there are currently other proton SFRT modalities under investigation. Proton minibeam radiotherapy (pMBRT) had drawn attention due to its striking normal-tissue sparing in preclinical brain/skin models and early patient-specific planning studies using thick multi-slit collimators at the nozzle exit (21–24). However, pMBRT remains largely preclinical because maintaining PVDR is depth-limited and challenging for deep tumors (e.g., PVDR ≈8–12 at ~20 mm, but ≈1.1–1.6 by ~80–90 mm of depth in tissue) (25). Also, pMBRT clinical delivery either relies on mechanical collimation which reduces efficiency and adds neutron production, or requires an optimized/new nozzle since current PBS nozzles are not suitable for magnetic minibeam generation (26–31). These limitations and sensitivity to motion for sub-millimeter patterns make pMBRT less immediately deployable for deep, moving abdominal targets like the liver (4, 24). In contrast, our PBS-based IMPT-LRT strategy is directly analogous to photon LRT (with growing clinical experience), uses standard hardware and TPS, integrates with robust optimization for motion/range, and is therefore more clinically applicable in the near term.

From a translational perspective, enhanced vertex and valley dose ratio and lower liver dose, support several clinical pathways: (i) single−fraction IMPT−LRT as a boost preceding or following conventionally fractionated EBRT; (ii) multi−fraction IMPT−LRT (e.g., 3–5 fractions) to balance biologic effectiveness and OAR recovery; and (iii) selective dose escalation for very large GTVs where photon lattice plans struggle to keep mean liver dose and central hepatobiliary tract within dose constraints. These strategies are consistent with emerging SFRT consensus documents and proton LRT planning reports emphasizing standardized vertex/valley endpoints (VTVH D80, VTVLmean, PVDR-like ratios) and robust delivery practices (range/setup robustness, gating/DIBH, repainting).

Our work focused on bulky liver cancer due to the complexity of treating this disease, as well as recent clinical results with liver LRT that show it is an emerging treatment approach. A 2023 systematic review concluded that LRT appears safe, with heterogeneous but promising efficacy signals across multiple treatment sites, while emphasizing the need for standardized endpoints and prospective data specific to the liver (32). For hepatocellular carcinoma (HCC), dosimetric feasibility work shows that VMAT-LRT can escalate intratumoral dose when conventional SBRT is OAR-limited, supporting the rationale for spatially modulated boosts in challenging hepatic presentations (8, 33). Clinically, a recent liver-focused case demonstrated rapid palliation and marked volumetric response after single-fraction 16 Gy LRT delivered to ~1% of the metastatic liver burden while keeping the mean liver dose ≈3 Gy and without acute toxicity, highlighting the potential of LRT to relieve symptoms at very low integral dose (15). Considering these circumstances, our PBS proton LRT workflow is designed to enhance valley sparing and lower non-target liver dose relative to VMAT-LRT, which should lead to better protection of functional parenchyma and facilitate dose intensification or fraction reduction in large hepatic targets. Mechanistically, improved valley sparing may better protect functional liver parenchyma, potentially lowering the risk of RILD when combined with following EBRT (1, 5, 34). At the same time, higher vertex doses could enhance intratumoral cytotoxicity and immune priming in heterogeneous hypoxic tumors (35–37). Our findings therefore provide a dosimetric foundation for protocol development in hepatic SFRT/LRT that prospectively measures liver function (e.g., ICG, HIDA, or SPECT/PET) alongside clinical toxicity and local control.

Delivery considerations are paramount for the liver. Proton PBS delivery is more sensitive to interplay effect due to respiration and peristalsis motions, and proton range uncertainty comparing with photon modalities. As a result, proper planning approaches and motion management strategies need to be established for IMPT-LRT to mitigate setup uncertainties, anatomical variations (from intra- and inter-fractional motion), and range uncertainties. In this study, inspiration breath hold gating was used during initial simulation for the patients included in this study. During planning, a 3-mm setup and 3.5% range uncertainty parameters were used in robust optimization, which is consistent with our clinical stereotactic body radiation therapy treatments that utilize protons. The 3mm setup uncertainty takes into account of patient setup variation and spot positioning accuracy. For liver stereotactic treatment, CT-on-Rails (CTOR) which has superior imaging quality than CBCT will be used during patient setup to minimize the setup error. Spot position accuracy is checked daily and monitored by the ProBeat system during treatment, where an averaged 1mm accuracy is expected. Before implementing in clinic, End-to-end tests will be conducted to evaluate the efficiency of the 3mm setup uncertainty.

This study had several limitations. First, this study used a planning−only study design (lack of delivery QA). The vertex integrity and valley sparing could be compromised during treatment due to variation of spot positioning, range uncertainties, patient’s position variations. End-to-end tests would be needed to establish the treatment protocol. Second, we used a relative biological effectiveness (RBE) factor of 1.1 for proton therapy, which may not sufficiently capture high−dose−per−fraction biology. We did not perform organ−specific statistical testing or subgroup analyses by GTV size. Nevertheless, the consistency of valley sparing across all cases and the magnitude of the PVDR−like improvements support the generalizability of the core finding. The second limitation is lack of strategy for robustness evaluation. During our clinical implementation of IMPT SFRT, for each candidate plan we will compute robustness scenarios including standard setup and range uncertainties and, critically, motion-specific scenarios, then assess three SFRT metrics per scenario: peak coverage within VTVH (e.g., D95), valley dose in the valley volume adjacent to the relevant OAR interface, and the resulting PVDR. For free-breathing deliveries, robustness will explicitly evaluate dose calculated on scans of T0 and T50; for DIBH, we will propagate the plan across multiple repeat breath-hold CTs acquired at simulation to capture breath-hold variability. We will summarize results as both scenario-average and scenario-minimum (“worst-case”) values, and reported as PVDR_robust, peak-coverage robust, and valley-dose robust, and require that all meet predefined acceptance thresholds, with particular emphasis on preserving PVDR_robust at the tumor–OAR interface. When thresholds are not met, we will iterate geometry and delivery (vertex size/placement, repainting, gating/beam timing, or modest beam re-arrangement. The third limitation is that this study only focuses on liver tumors. We focus on bulky liver tumors because (i) hepatic toxicity tightly correlates with the volume of uninvolved liver receiving low–intermediate dose, making valley sparing particularly impactful; (ii) tumors often in close proximity to stomach/duodenum/bile ducts, where sharper dose fall-off may reduce OAR toxicities; (iii) the liver’s respiratory motion is manageable with 4D robust optimization, gating/repainting, fiducial/surface guidance or breath-hold techniques; and (iv) liver IGRT is mature yet imperfect, so range-modulated valleys may better protect parenchyma under uncertainty. This setting offers a clean readout of proton IMPT LRT’s dosimetric advantage. Future work will evaluate proton IMPT LRT in additional sites and histologies that photon LRT has shown feasibility and early clinical signal, which includes non-small cell lung cancer (NSCLC), head and neck, gynecologic (bulky cervix), and soft-tissue sarcoma. In voluminous NSCLC, retrospective data suggest safety and tumor response (38). Palliative head-and-neck series report symptom relief with acceptable toxicity (39). Prospective multi-site data (LITE SABR M1) support short-term safety in large tumors, and time-resolved case series show rapid palliation across sites (40, 41). For gynecologic disease, far-advanced cervical cancer has demonstrated high metabolic responses with good tolerance after LRT (42). In sarcoma, neoadjuvant extremity cases show technical feasibility of integrating high-dose lattice with standard RT (43). These experiences motivate site-specific proton LRT protocols to test whether enhanced valley-sparing can further reduce normal-tissue dose while preserving (or improving) clinical benefit.

Future work will include LET optimization to further enhance the dose contrast in vertex and valley, and thorough study of organ-specific studies for different lattice construction and different planning techniques, as well as understanding the RBE effects of proton LRT in clinical treatment planning and delivery.

Moreover, only DIBH was used as motion management method in this planning study. Based on our experience in proton treatment, respiratory excursions over 5 mm produce phase-dependent hot/cold spots between T0 (end-inspiration) and T50. This effect would amplify and erode planned PVDR in single-fraction SFRT, where minimal temporal averaging leaves interplay unmitigated. We therefore will explore a threshold-based policy for future study: for motion less than 5mm, we create the plan using free breathing CT scan with T0/T50 explicitly included in robust optimization and apply layered repainting during delivery. For motion equal to or higher than 5mm, we treat with deep-inspiration breath-hold (DIBH) and, to account for breath-hold variability between simulation and treatment, acquire more than 3 repeat DIBH CTs at simulation and include all repeats in robustness optimization. If DIBH proves infeasible, we revert to amplitude/phase gating with compression and increase repainting to further mitigate interplay (44, 45). As a future direction, we will prospectively benchmark PVDR erosion vs motion amplitude, test whether multi-CT DIBH planning stabilizes valley dose relative to single-CT approaches, and define adaptive re-simulation triggers (e.g., >2–3 mm drift in observed motion or breath-hold level). Recent developments in proton SFRT include robust multi-field planning strategies (e.g., primary plus robust complementary beams) and proton arc-based lattice (ARC/SPArc) are concepts that seek to balance vertex intensification with valley suppression and OAR sparing (19, 20, 30). Motion management strategies and planning studies could be explored with proton ARC/SPArc lattice concepts to further reduce the valley dose and minimize entrance dose, albeit at the slight increase of integral dose.

## Conclusion

In summary, proton-based IMPT−LRT is feasible and shows significant dosimetric advantages over traditional photon-based VMAT−LRT for liver tumors in this paired planning study. IMPT−LRT provided superior valley sparing and higher vertex coverage than VMAT−LRT for large liver tumors, with comparable PRV hotspots under a shared lattice template. In terms of plan quality and dosimetry, our results establish IMPT-LRT as a superior modality to VMAT-based LRT with deeper valleys, stronger peaks, same OAR safety, and provide a quantitative basis for prospective clinical implementation (e.g., boost strategies or multi-fraction LRT).

## Data Availability

The raw data supporting the conclusions of this article will be made available by the authors, without undue reservation.
